# Assessment of Genetic Fidelity in *Rauvolfia serpentina* Plantlets Grown from Synthetic (Encapsulated) Seeds Following *in Vitro* Storage at 4 °C

**DOI:** 10.3390/molecules17055050

**Published:** 2012-05-03

**Authors:** Mohammad Faisal, Abdulrahman A. Alatar, Naseem Ahmad, Mohammad Anis, Ahmad K. Hegazy

**Affiliations:** 1 Department of Botany & Microbiology, College of Science, King Saud University, P.O. Box 2455, Riyadh 11451, Saudi Arabia; 2 Plant Biotechnology Laboratory, Department of Botany, Aligarh Muslim University, Aligarh-202 002, India

**Keywords:** acclimatization, encapsulation, short-term storage, plant regeneration, molecular marker

## Abstract

An efficient method was developed for plant regeneration and establishment from alginate encapsulated synthetic seeds of *Rauvolfia serpentina*. Synthetic seeds were produced using *in vitro* proliferated microshoots upon complexation of 3% sodium alginate prepared in Llyod and McCown woody plant medium (WPM) and 100 mM calcium chloride. Re-growth ability of encapsulated nodal segments was evaluated after storage at 4 °C for 0, 1, 2, 4, 6 and 8 weeks and compared with non-encapsulated buds. Effects of different media *viz*; Murashige and Skoog medium; Lloyd and McCown woody Plant medium, Gamborg’s B5 medium and Schenk and Hildebrandt medium was also investigated for conversion into plantlets. The maximum frequency of conversion into plantlets from encapsulated nodal segments stored at 4 °C for 4 weeks was achieved on woody plant medium supplement with 5.0 μM BA and 1.0 μM NAA. Rooting in plantlets was achieved in half-strength Murashige and Skoog liquid medium containing 0.5 μM indole-3-acetic acid (IAA) on filter paper bridges. Plantlets obtained from stored synseeds were hardened, established successfully *ex vitro* and were morphologically similar to each other as well as their mother plant. The genetic fidelity of *Rauvolfia* clones raised from synthetic seeds following four weeks of storage at 4 °C were assessed by using random amplified polymorphic DNA (RAPD) and inter-simple sequence repeat (ISSR) markers. All the RAPD and ISSR profiles from generated plantlets were monomorphic and comparable to the mother plant, which confirms the genetic stability among the clones. This synseed protocol could be useful for establishing a particular system for conservation, short-term storage and production of genetically identical and stable plants before it is released for commercial purposes.

## 1. Introduction

*Rauvolfia serpentina* Benth. (Apocynaceae), commonly known as Indian snakeroot or Sarpagandha, is indigenous to India and other tropical countries of Asia. The *Serpentina* plant has drawn special attention all over the World in the pharmaceutical field and holds an important position because of the presence of number of bioactive chemicals it containes, including ajmaline, deserpidine, rescinnamine, serpentinine, and yohimbine. Reserpine is a potent alkaloid first isolated from this plant which is being widely used as an antihypertensive. This herbal plant is used as medicine for high blood pressure, insomnia, anxiety and other disorders of the central epilepsy [1]. A major part of the commercial supply of the drug used in U.S.A. and European countries originates from India, Pakistan, Sri Lanka, Burma and Thailand, with India being a major supplier. Poor seed viability, low seed germination rate, and low vegetative propagation rate through rooted cuttings has hampered large scale commercial cultivation of *R. serpentina* through conventional modes and over exploitation of the natural resources has led to listing of this species as “endangered” by the International Union for Conservation of Nature and Natural Resources (IUCN) [2]. Under these circumstances, propagation through biotechnological approaches of this potential drug producing plant has assumed importance. 

Production of synthetic seed has provided new opportunities in plant biotechnology. The alginate encapsulation technique is designed to combine the advantages of clonal propagation with those of seeds propagation and storage [3,4]. Although many reports are available on the utilization of synthetic seeds for micropropagation and conservation of various medicinal plant species [5,6,7,8,9,10,11]. The genetic stability of synthetic seed-derived plantlets remains relatively unknown with the exception of some recent reports on *Ananus comosus* [12], *Cineraria maritima* [13] and *Picrorhiza kurrooa* [14]. True-to-type clonal fidelity is one of the most important pre-requisites in the *in vitro* propagation of crop species. The occurrence of cryptic genetic defects arising via somaclonal variation in the regenerates can seriously limit the broader utility of the micropropagation system [15]. It is, therefore, imperative to establish genetic uniformity of synthetic seed derived plantlets to suggest the quality of the plantlets for its commercial utility. Polymerase chain reaction (PCR)-based techniques such as random amplified polymorphic DNA (RAPD) and inter-simple sequence repeat (ISSR) are immensely useful in establishing the genetic stability of *in vitro*-regenerated plantlets in many crop species [16,17]. RAPD and ISSR markers are very simple, fast, cost-effective, highly discriminative and reliable. They require only a small quantity of DNA sample and they do not need any prior sequence information to design the primer. They do not use radioactive probes as in restriction fragment length polymorphism (RFLP) [16]; thus, they are suitable for the assessment of the genetic fidelity of in vitro-raised clones. However, no studies on genetic fidelity of synseed-derived plantlets of *R. serpentina* have been reported.

We encapsulated individual microshoots of *R. serpentina* in sodium alginate beads and standardized a protocol for *in vitro* regeneration and short-term storage to ensure steady supply and exchange of quality plant materials. To guarantee that synthetic seed technology will indeed conserve the micropropagated propagules of *R. serpentina*, following their conversion from encapsulated nodal segments, the genetic fidelity of the synthetic seed-derived plantlets is assessed using RAPD and ISSR markers. 

## 2. Results and Discussion

**Figure 1:**
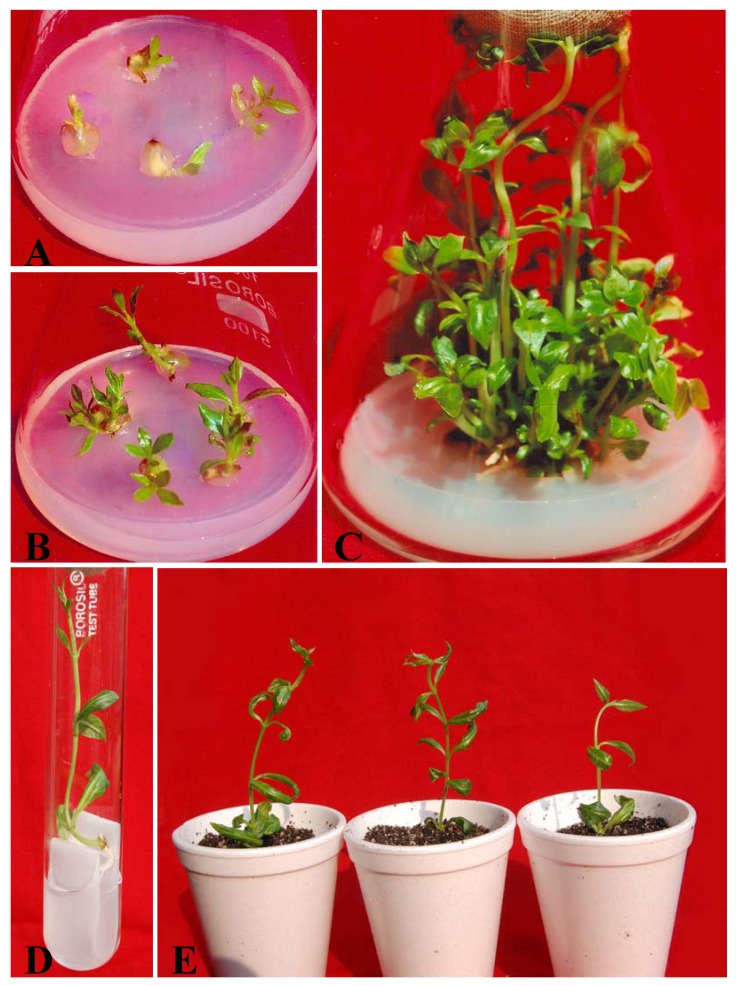
Plant regeneration from synthetic seeds of *R. serpentina* formed by the encapsulation of nodal segments in 3% (w/v) sodium alginate and 100 mM calcium chloride; A. Shoot formation from encapsulated nodal segments on WPM + 5.0 μM BA + 1.0 μM NAA after 2 weeks of culture; B. 3 weeks old culture showing shoot formation from synthetic stored at 4 °C for 4 weeks. Shoot multiplication from encapsulated nodal segments on WPM + 5.0 μM BA + 1.0 μM NAA after 8 weeks of culture; D. Rooted synseed derived plantlets. E. Acclimatized plantlets derived from synthetic seeds.

Nodal segments encapsulated in 3% (w/v) sodium alginate and 100 mM calcium chloride stored at 4 °C for 1, 2, 4, 6 or 8, placed on woody plant medium supplemented with 5.0 µM BA and 1.0 µM NAA showed emergence of shoots after 2 weeks of incubation (Figure 1A,B). After four weeks of storage at 4 °C, the percentage conversion of encapsulated nodal segments into complete plantlets was 80%, whereas about 21% of non-encapsulated nodal segments produced plantlets. The alginate matrix, supplemented with the necessary ingredients, served as an artificial endosperm, thereby providing nutrients to the encapsulated explants for re-growth [18,19]. Antonietta *et al*. [19] reported that a synthetic endosperm should contain nutrients and a carbon source for germination and conversion. The conversion into plantlets from encapsulated nodal segments decreased as the period of storage increased beyond four weeks (Table 1). The decline in the conversion response could be attributed to oxygen deficiency in the encapsulated beads, or to a loss of moisture due to partial desiccation during storage [9,20].

**Figure 2:**
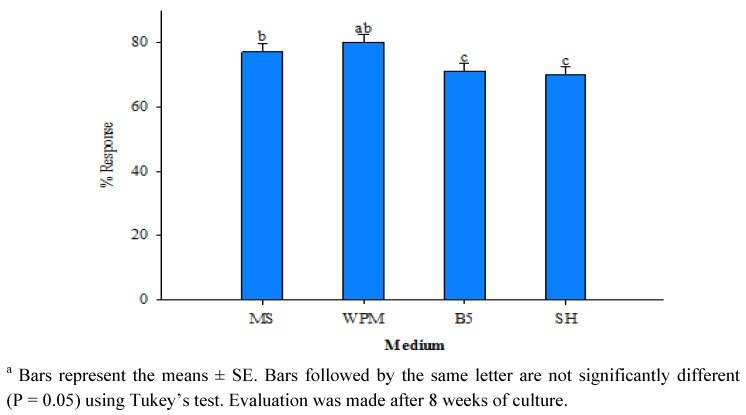
Effect of different medium supplemented with 5.0 µM BA and 1.0 µM NAA on the conversion of encapsulated nodal segments of *R. serpentina* after 4 weeks of storage at 4 °C *^a^*.

Different basal media MS, WPM, B5 and SH with 5.0 µM BA and 1.0 µM NAA were examined for inducing maximum conversion into shoots from encapsulated buds following storage for four weeks at 4 °C. Results revealed significant (P = 0.05) performance on woody plant medium to give maximum response of conversion of encapsulated buds into multiple shoot (Figure 2). After eight weeks of culture well-developed shoot were observed on this medium (Figure 1C). However, the lowest frequency of shoot formation from encapsulated bead was observed in SH medium. The data from our experiment with cold-stored encapsulated nodal segments were in agreement with the study of Faisal *et al*. [21] concerning the rates of encapsulated segments with axillary buds in *Rauvolfia tetraphylla* stored at 4 °C. Similarly, *Eclipta alba* encapsulated buds cold-stored for 60 days were found to display better performance and conversion indices than non-encapsulated one [22]. The regenerated shoots were rooted in half-strength MS liquid medium containing 0.5 μM IAA on filter paper bridges (Figure 1D). Plantlets with 4–5 fully expanded leaves and well-developed roots were successfully hardened off inside the growth room in planting substrates for 4 weeks and were eventually established in natural soil (Figure 1E). Of the three different types of planting substrate examined, percentage survival of the plantlets was highest (90%) in soil-rite (Table 2) and lowest (53.3%) in garden soil. About 90% of the micropropagated plants survived following transfer from soil-rite to natural soil and did not show any detectable variation in respect to morphology or growth characteristics. This observation is in agreement with several earlier findings [6,9,13].

**Table 1:** Effect of different duration of storage at 4 °C on the conversion of encapsulated and non-encapsulated nodal segments *R. serpentina* after 8 weeks of culture on woody plant medium supplemented with 5.0µM BA and 1.0 µM NAA *^a^*.

Storage duration (Weeks)	Encapsulated buds	Non-encapsulated buds
0	91.6 ± 2.7 ^a^	93.0 ± 3.0 ^a^
1	85.0 ± 2.3 ^ab^	57.0 ± 2.6 ^b^
2	81.4 ± 2.6 ^b^	40.2 ± 2.3 ^c^
4	80.0 ± 2.3 ^b^	21.0 ± 1.8 ^d^
6	57.3 ± 2.0 ^c^	17.1 ± 1.6 ^d^
8	50.0 ± 1.8 ^d^	7.0 ± 1.1 ^e^

*^a^* Values represent the means ± SE. Means followed by the same letter within the column are not significantly different (P = 0.05) using Tukey’s test. Evaluation was made after 8 weeks of culture.

**Table 2:** Effect of different planting substrates for hardening off synseed-raised plantlets of *Rauvolfia serpentina ^a^.*

Planting substrate	Number of plants transferred	Number of plants survived	Plant survival (%)
Garden soil	30	16	53.3
Soil-rite	30	27	90.0
Vermiculite	30	25	76.6

^a^ Data were recorded after four weeks of transfer to planting substrates.

**Figure 3:**
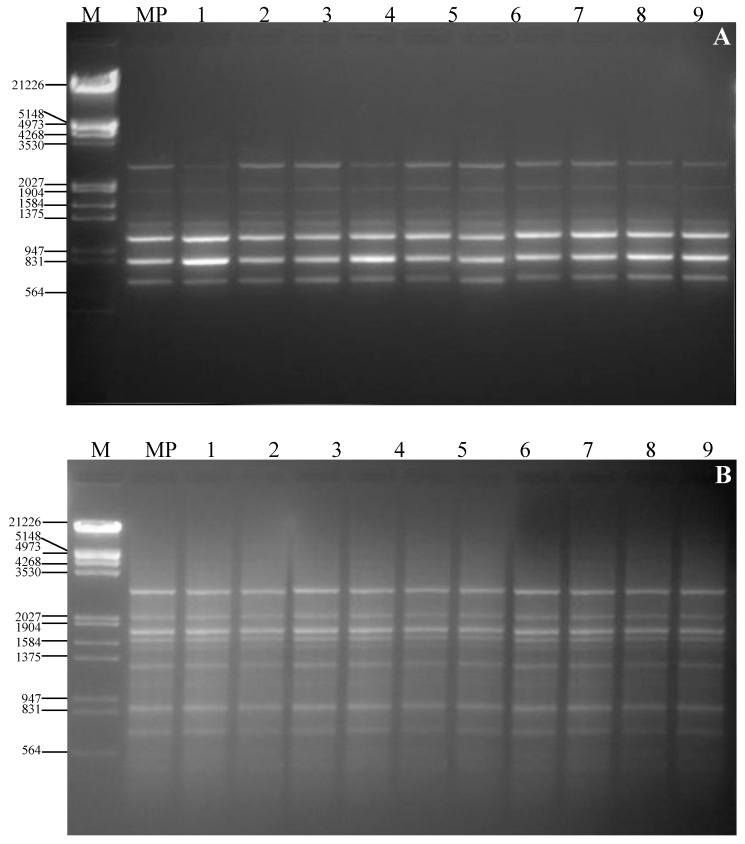
Agarose gel electrophoresis of RAPD and ISSR products of synseed-raised *Rauvolfia serpentina* plantlets and mother line obtained with primer OPA-11 (A) and ISSR-03 (B). Lanes 1–9, regenerants; Lane MP=mother plant; lane M = Lambda DNA/Eco RI + HindIII marker indicated in bp.

For RAPD analysis 20 primers were used for initial screening with the mother plant of *R. serpentina* and 19 RAPD primers gave clear and reproducible bands. The number of scorable bands for each RAPD primer varied from 2 (OPA-1) to 7 (OPA-12) (Table 3). The 19 RAPD primers produced 160 distinct and scorable bands, with an average of 8.4 bands per primer). No polymorphism was detected during the RAPD analysis of *in vitro*-raised clones (Figure 3). All seven ISSR primers used in the initial screening produced clear and reproducible bands. The optimum annealing temperature for ISSR markers varied from 45.7 to 49.0 °C (Table 4). The number of scorable bands for each primer varied from eight (ISSR-01) to 17 (ISSR-06), with an average of 11.4 bands per primer. All banding profiles from micropropagated plants were monomorphic and similar to those of the mother plant (Figure 4). Our results corroborate with the earlier reports on genetic stability of synthetic seed derived plantlets of *Ananus comosus* [12], *Cineraria maritime* [13] and *Picrorhiza kurrooa* [14].

**Table 3:** List of RAPD primers used to verify the genetic fidelity of micropropagated plantlets of *Rauvolfia serpentina*.

S. No.	Name of primers	Primer sequence (5´ – 3´)	Number of bands
1	OPA-01	CAGGCCCTTC	12
2	OPA-02	TGCCGAGCTG	12
3	OPA-03	AGTCAGCCAC	7
4	OPA-04	AATCGGGCTG	14
5	OPA-05	AGGGGTCTTG	8
6	OPA-06	GGTCCCTGAC	1
7	OPA-07	GAAACGGGTG	7
8	OPA-08	GTGACGTAGG	5
9	OPA-09	GGGTAACGCC	10
10	OPA-10	GTGATCGCAG	13
11	OPA-11	CAATCGCCGT	6
12	OPA-12	TCGGCGATAG	7
13	OPA-13	CAGCACCCAC	15
14	OPA-14	TCTGTGCTGG	12
15	OPA-15	TTCCGAACCC	5
16	OPA-16	AGCCAGCGAA	0
17	OPA-17	GACCGCTTGT	3
18	OPA-18	AGGTGACCGT	11
19	OPA-19	CAAACGTCGG	4
20	OPA-20	GTTGCGATCC	8

**Table 4:** List of ISSR primers used to verify the genetic fidelity of micropropagated plantlets of *Rauvolfia serpentina ^a^*.

S. No.	Name of primers	Primer sequence (5′ – 3′)	Annealing temperature °C	Number of bands
1	ISSR-01	ACA CAC ACA CAC ACA CT	45.7	8
2	ISSR-02	ACA CAC ACA CAC ACA CG	49.0	12
3	ISSR-03	AGA GAG AGA GAG AGA GYT	49.0	12
4	ISSR-04	GAG AGA GAG AGA GAG AYC	49.0	13
5	ISSR-05	ACA CAC ACA CAC ACA CYT	49.0	15
6	ISSR-06	DBD ACA CAC ACA CAC AC	45.7	17
7	ISSR-07	HVH TGT GTG TGT GTG TG	45.7	13

^a^ Y = (CT), B = (CGT) (*i.e*. not A), D = (AGT) (*i.e*. not C), H = (ACT) (*i.e*. not G), V = (ACG) (*i.e*. not T).

The two PCR-based techniques, RAPD and ISSR, were used to test clonal fidelity because of their simplicity and cost-effectiveness. The use of two markers, which amplify different regions of the genome, allows better chances for the identification of genetic variation [23]. In this study the number of bands generated per primer was greater in ISSR (11.4) than RAPD (8.4). These differences could possibly be due to the high melting temperature for the ISSR primers, which permits much more stringent annealing conditions and, consequently, more specific and reproducible amplification. Devarumath *et al*. [24] also revealed that ISSR fingerprints detected more polymorphic loci than RAPD fingerprinting. 

## 3. Experimental

### 3.1. Plant Material and Explant Source

Nodal segments of *R. serpentina*, collected from a one-year-old field-grown plant, were washed thoroughly under running tap water for 30 min, then immersed in 5% (v/v) detergent (LaboleneTM; Qualigens, Mumbai, India) for 5 min. After through washing, their surfaces were sterilized with 0.1% (w/v) HgCl_2_ for 3 min and finally rinsed 4–5 times with sterile distilled water to remove all traces of sterilant. The sterilized nodal segments were cultured on woody plant medium [25] supplemented with 5.0 μM BA and 1.0 μM NAA. Nodal segments, approx. 3–5 mm long, excised from *in vitro* proliferating cultures were encapsulated aseptically. 

### 3.2. Encapsulation and Low Temperature Storage

For encapsulation 3% (w/v) sodium alginate 100 mM calcium chloride (CaCl_2_·2H_2_O) solutions were prepared in liquid woody plant medium. Both the gel matrix and the complexing agent were autoclaved at 121 °C for 20 min under 15 kPa. First the nodal segments were mixed into an alginate solution, and then dropped into calcium chloride solutions for at least 30 min to achieve gel complexation. After 30 min, the alginate beads were collected and rinsed with sterile distilled water to remove excess calcium chloride. Encapsulated and non-encapsulated nodal segments were transferred to 100 × 15 mm Petri dishes containing water-agar medium and stored in a refrigerator at 4 °C. The media were solidified with 0.8% (w/v) agar before being autoclaved at 121 °C for 20 min under 15 kPa. Five different low temperature storage periods (1, 2, 4, 6 or 8 weeks) were evaluated for their effect on regeneration. 

#### Plant regeneration and culture condition

After each period of storage at 4 °C, encapsulated and non-encapsulated nodal segments were cultured on woody plant medium containing 5.0 μM BA and 1.0 μM NAA for conversion into plantlets. Different basal medium were also examined and compared to detect best medium suitable for maximum shoot regeneration from encapsulated nodal segments following cold storage at 4 °C for four weeks. Murashige and Skoog [26] (MS), WPM, Gamborg *et al*., [27] (B_5_) and Schenk and Hildebrandt, [28] (SH) medium were used with 5.0 µM BA and 1.0 µM NAA. After sprouting, each encapsulated bud was transferred to a 250 mL culture flask containing WPM with 5.0 µM BA and 1.0 µM NAA. The regenerated shoots were excised and transferred individually to MS medium containing 0.5 μM IAA (indole-3-acetic acid) for root development. The media were solidified with 0.8% (w/v) agar and the pH was adjusted to 5.8 with 0.1 M NaOH before being autoclaved at 121 °C for 20 min under 15 kPa pressure. All cultures were maintained at 24° ± 2 °C under a 16 h photoperiod with a photosynthetic photon flux density of 50 μmoles m^–2^ s^–1^, provided by cool-white fluorescent lamps. 

### 3.3. Acclimatization

Rooted plantlets were removed from the culture medium, washed gently under running tap water and transferred to plastic pots containing sterile garden soil, soil-rite (75% Irish peat moss and 25% horticulture grade expanded perlite) or vermiculite (Keltech Energies Ltd., Bangalore, India) under diffuse light (16:8 h photoperiod) conditions. Potted plantlets were covered with a transparent polythene membrane to ensure high humidity and watered every three days with half-strength MS salt solution for two weeks. Polythene membranes were opened after two weeks in order to acclimatize plants to field conditions. After four weeks, acclimatized plants were transferred to pots containing normal soil and maintained in a greenhouse under normal day length conditions. 

### 3.4. Genomic DNA Extraction and PCR Amplification

Clonal fidelity of synthetic seeds raised clones was tested using RAPD and ISSR markers. For this purpose, 10 hardened plants, raised from synseed (stored at 4 °C for 4 weeks) were chosen randomly from the population and compared with the mother plant from which the explants were taken. Total genomic DNA of the mother plant and *in vitro*-raised clones was extracted from young leaf tissues by using the modified cetyltrimethyl ammonium bromide (CTAB) method described by Doyle and Doyle [29], as modified by Weising *et al*. [30]. Purified total DNA was quantified and its quality verified by spectrophotometry (UV 1700-PharmaSpec, Shimadzu, Tokyo, Japan). Regenerated plantlets were tested for clonal fidelity using 10 RAPD primers (Table 3) and seven ISSR primers (Table 4) for their unambiguous and reproducible band patterns.

PCR amplifications were carried out as described by Willams *et al*. [31] in a total volume of 25 µL containing 25 ng total DNA, 1 μL PCR buffer (Fermentas GmbH, Germany), 2.0 mM MgCl_2_, 200 mM dNTPs, 1 μL 10-mer oligode- oxynucleotide RAPD (Operon Technologies Inc., Germany) or ISSR (Bangalore Genei, Bangalore, India) primer and 1 unit (U) Taq DNA polymerase (Fermentas). The PCR amplification was performed using Thermal cycler (T-Gradient Biometra, Gottingen, Germany). For RAPD analysis, PCR temperature profiles were used as initial DNA denaturation at 94 °C for 5 min followed by 40 cycles at 94 °C for 1 min, 35 °C for 1 min, and 72 °C for 2 min. Final cycle at 72 °C for 7 min was also performed. For ISSR markers, PCR reactions were performed with initial DNA denaturation at 94 °C for 5 min followed by 35 cycles at 94 °C (1 min) for DNA denaturation, 45.7 to 49 °C (1 min) for primer annealing (Table-4), 72 °C (2 min) for primer extension and final extension at 72 °C for 7 min. All the amplified PCR products obtained from RAPD and ISSR markers were resolved by electrophoresis on 1.4% agarose gel for 3 h in 1× TBE buffer, stained with ethidium bromide, and the photographs taken using Gel Documentation System (Gel-Doc EZ System, Bio-Rad, USA).

### 3.5. Statistical Analysis

All the experiments were conducted in a completely randomized block design (CRD) with ten explants per treatment and each treatment was repeated three times. The results are expressed as a mean ± SE of three independent experiments. The cultures were observed periodically and morphological changes were recorded at regular intervals. Data on percentage of conversion of encapsulated nodal segments were recorded after eight weeks of culture. Hardening of plantlets was evaluated after four weeks of transfer to pots and percentage of survival of plantlets was recorded for different potting mixtures. All the data were subjected to analysis of variance (ANOVA) followed by Tukey’s test at P = 0.05 using SPSS software version 20 (SPSS Inc., Chicago, IL, USA). 

## 4. Conclusions

In conclusion, we have established an efficient system for plant regeneration from encapsulated nodal segments of *R. tetraphylla*, which not only provides an alternative viable system for storage and clonal multiplication, but also explores the possibility of preserving the genetic stability of the selections or promotes true-to-type genotypes for exchange between national and international laboratories.
